# A Systematic Review of African Studies on Intimate Partner Violence against Pregnant Women: Prevalence and Risk Factors

**DOI:** 10.1371/journal.pone.0017591

**Published:** 2011-03-08

**Authors:** Simukai Shamu, Naeemah Abrahams, Marleen Temmerman, Alfred Musekiwa, Christina Zarowsky

**Affiliations:** 1 School of Public Health, University of the Western Cape, Cape Town, South Africa; 2 Gender and Health Unit, Medical Research Council Cape Town, Cape Town, South Africa; 3 International Centre for Reproductive Health, Ghent University, Ghent, Belgium; 4 Biostatistics Unit, Medical Research Council, Cape Town, South Africa; Indiana University, United States of America

## Abstract

**Background:**

Intimate partner violence (IPV) is very high in Africa. However, information obtained from the increasing number of African studies on IPV among pregnant women has not been scientifically analyzed. This paper presents a systematic review summing up the evidence from African studies on IPV prevalence and risk factors among pregnant women.

**Methods:**

A key-word defined search of various electronic databases, specific journals and reference lists on IPV prevalence and risk factors during pregnancy resulted in 19 peer-reviewed journal articles which matched our inclusion criteria. Quantitative articles about pregnant women from Africa published in English between 2000 and 2010 were reviewed. At least two reviewers assessed each paper for quality and content. We conducted meta-analysis of prevalence data and reported odds ratios of risk factors.

**Results:**

The prevalence of IPV during pregnancy ranges from 2% to 57% (n = 13 studies) with meta-analysis yielding an overall prevalence of 15.23% (95% CI: 14.38 to 16.08%). After adjustment for known confounders, five studies retained significant associations between HIV and IPV during pregnancy (OR1.48–3.10). Five studies demonstrated strong evidence that a history of violence is significantly associated with IPV in pregnancy and alcohol abuse by a partner also increases a woman's chances of being abused during pregnancy (OR 2.89–11.60). Other risk factors include risky sexual behaviours, low socioeconomic status and young age.

**Conclusion:**

The prevalence of IPV among pregnant women in Africa is one of the highest reported globally. The major risk factors included HIV infection, history of violence and alcohol and drug use. This evidence points to the importance of further research to both better understand IPV during pregnancy and feed into interventions in reproductive health services to prevent and minimize the impact of such violence.

## Introduction

Women of reproductive age are more vulnerable to abuse by intimate partners than by any other perpetrator [1]. Prevalence of Intimate Partner Violence (IPV) against pregnant women differs across populations globally with rates reported to range from 0.9 to 20.1% in a systematic review that included 13 studies conducted before 1996 [2]. A second review of 18 studies reported prevalence of physical violence against pregnant women ranging between 0.9% and 30% [3]. Only six studies were from developing countries (reporting a range from 1.3% to 12.6%) in which only one was from Africa. Whilst it can be argued that with the passage of time, more defined and comprehensive measures were used to measure violence more accurately and with greater disclosure, very broad prevalence ranges persist as reflected in the 2010 review [3] compared to the 1996 review [2]. The low rates of violence reported in studies from developing countries in the 2010 review cannot be interpreted without special focus on context and risk factors and that further inquiry focusing on Africa in particular is needed. In addition both reviews did not cover all African databases, journals and archives and these findings cannot be generalized to African populations given the socio-cultural, political, economic and gender power differences. More recent data from the World Health Organisation Multicounty study [4] reported prevalence estimates of between 1% and 28% for the ten participating countries with the highest prevalences reported from the two African countries: Ethiopia and Tanzania [5].

There are significant negative maternal and child health outcomes associated with violence against pregnant women which are directly linked to Millennium Development Goals (MDGs) number 4 and 5 to reduce child mortality and improve maternal health as well as MDG 3 to promote gender equality and empowerment of women [6]. These negative health outcomes include pregnancy loss, preterm labour, pregnancy complications, hypertension, delivering low birth weight, physical injuries and stress [4], [7]. IPV has also been reported as a contributing cause of maternal deaths [8] and there is therefore need to synthesize information on risk factors from studies on abused pregnant women to quantify the problem and inform responses. Such information can help to advocate health interventions such as screening pregnant women for IPV to contribute to safe motherhood and healthy babies.

Pregnant women are at a higher risk of experiencing gender-based violence because they are more likely to be in relationships compared to non-pregnant population [3]. In addition, their age (15–49 years old) has also been identified as a higher risk group for IPV. Analyzing the evidence from studies on this population is critical for interventions since pregnancy related services provide excellent opportunities to assess the extent to which women experience abuse by partners and grant opportunities to assist and support them – all which would contribute to the meeting of the MGDs.

Many of the risk factors for IPV during pregnancy have also been identified generally in IPV studies among women [9]. The socio-demographic risk factors reported by Taillieu and Brownridge [3] included being young or adolescent; single marital status; separated or divorced during pregnancy; belonging to ethnic minorities and low educational status. For example, less education may translate to limited opportunities and increases economic vulnerability leading to some women being abused by partners who may be economically more powerful than them. Adolescents who are usually less mature to handle relationships or marriages may also be economically vulnerable and at risk of submitting to male power and abuse. Other risk factors identified included increased substance and drug use [3], [10] as intoxication may lead to irresponsible behaviour such as violence. Perpetrator characteristics associated with IPV during pregnancy include male controlling behavior and having economic power [11], [12]. In Africa, feminization of poverty means that many poor women often rely on their partners for household maintenance and pregnancy care. Men exploit this economic vulnerability by abusing their partners. Pregnancy related factors found to be associated with experiencing IPV during pregnancy include unintended pregnancy, late entry into care and inadequate antenatal care [10], [13]. Unintended and unplanned pregnancy is usually blamed on the female partner and could be punished by divorce or threats to divorce in some parts of Africa. Men fear responsibilities which go with a pregnancy and therefore less likely to sanction a pregnancy if they were not prepared for it [14]. This is possibly due to male domination and control of female partners which starts upon marriage when the control of female sexuality is transferred from the father to the husband which in many African traditional cultures is officialised by sending marriage payments [15]. The control of household income which usually rests with male partners may influence late or inadequate prenatal entry. Abuse in childhood has been found to be associated with IPV among women in general [16], [17], [18] but information among pregnant women remains to be reviewed.

There are increasing studies from Africa that report on the relationship between HIV infection and IPV [19], [20], [21], [22]. In a review of literature on HIV and domestic violence, Kaye reported that violence against female partners increases when a female partner is known to be HIV positive [23]. Similarly, studies in Rwanda [24], Tanzania [25], and Kenya [1] have shown associations between HIV and IPV in a non-pregnant population; however a study in the USA had contrasting findings [26]. Potential ways in which HIV infection may be linked to intimate partner violence, based on studies mainly emerging from Africa include: physical vaginal trauma from forced sex; limited capability to negotiate safer sex due to partner violence or threat of it; violence following disclosure of a positive HIV result and perpetrators more likely to engage in risky sexual behavior [27].

### Research Question

Despite the fact that violence against women is reported as amongst the highest and severest in Africa compared to other continents [4], [28], evidence from a recent systematic review on domestic violence, which excluded studies among pregnant women, showed that relatively few studies and publications emerged from Africa compared to North America and Europe [29]. Amongst the 134 studies reviewed only 11% were conducted in Africa. Given the high prevalence of IPV in Africa and the increasing number of good scientific enquiry on violence against pregnant women in Africa, a systematic analysis would help to inform both research and action on the continent. The evidence from a systematic review could be used for development of policies for prevention of IPV, advocacy programmes for IPV in general and during pregnancy. At a service level it could influence health workers to screen pregnant women for IPV and lead to effective referrals and interventions.

### Purpose of the review

The aim of this systematic review was to systematically sum up the evidence from original empirical research conducted in Africa on prevalence and risk factors for IPV among pregnant women. The review also assesses the quality of the studies on IPV.

## Methods

### Search strategy

Searching of electronic databases using ebscohost was the primary way for obtaining peer reviewed journal articles in this review. A search of the Medline, Google scholar, Pubmed, SocIndex, Academic Search Premier, Family and Society Studies Worldwide, PsycArticles, Women's Studies International, Africa Wide Information databases was conducted to obtain articles on violence during the time of pregnancy. The search, which was conducted until January 2010, was restricted to articles published between January 2000 and January 2010 in all databases and journals searched. This period was chosen because studies only emerged from Africa from late 1990's and no systematic review for this continent has been conducted. Separate searches were conducted using the following key words: intimate partner violence, gender-based violence, violence against women, pregnant women, spousal violence, domestic violence, wife beating, wife abuse, spousal abuse, violence in pregnancy, violence and antenatal care, Africa, prevalence, risk factors, associations. Reference lists of the articles being reviewed were checked and relevant articles included. An independent hand search was conducted on specific African journals. Full text of some articles that only showed abstracts in the electronic databases or journals searched were obtained by emailing authors of the papers. The articles were checked for duplications in the different databases searched.

### Eligibility criteria

The eligibility criteria were: studies published between January 2000 and January 2010; articles based on original quantitative research results and conducted in any African country using any of the following study designs: cross sectional, cohort, case control, randomized controlled trial; articles published in English; all studies had to be peer reviewed in academic journals; studies had to include pregnant women (or mothers attending postnatal care within two months of giving birth); the women had to be the primary source of information and lastly articles had to focus on prevalence of IPV (physical, sexual and emotional) and/or risk factors for IPV among pregnant abused women. Intimate partners included past and current spouses, boyfriends, fiancés, whether married, cohabiting or dating. From all the studies that were included for systematic review, only those that reported overall prevalence of IPV were included in meta-analysis.

### Data collection process

Using a specially designed data extraction form, two reviewers independently extracted information from the papers. Data items included country, study design, sample size, response rate, target population, sampling method, tools used, case definition, interview type and outcomes from each study. Papers were examined to ensure that they do not display the same data set in different papers. If two articles were from the same data set but reporting on different variables both articles were considered. Where there was conflict in scoring between the reviewers, consensus was reached by three reviewers. Study authors were contacted in the case of unclear or missing data.

### Quality of studies and risk of bias

In order to assess the quality of studies and risk of bias, criteria developed by Alhabib et al [29] (2009) was adapted and applied. The following criteria was used: 1) Specification of the target population; 2) use of adequate sampling methods (eg random sampling); 3) adequate sample size (at least 300 participants); 4) adequate response rate (≥80%); 5) measurement with valid, tested instrument [eg Conflict Tactics Scale 2 (CTS2) [30], Abuse Assessment Screen (AAS)] [31]; 6) reporting confidence intervals or standard errors; 7) reported attempt to reduce observer or other forms of bias; 8) adjusted for confounding variables. Reviewers categorized instruments into CTS, AAS, the WHO questionnaire for measuring domestic violence against women [28] and lastly “own tool” where no known instrument was used. Where no values were provided in non-statistically significant relationships, we stated that the relationship was not statistically significant and that the *p*-value was not provided.

### Data analysis

There were two stages of data analysis. Firstly, for the analysis of prevalence of IPV, we conducted a fixed effect meta-analysis using STATA 11 [32] statistical software and results were presented using forest plots with prevalences and 95% confidence intervals. Heterogeneity between studies was assessed by using the I-square statistic [33] and by visually examining the forest plot for overlapping confidence intervals. As this revealed substantial heterogeneity, we decided not to use the pooled result from meta-analysis (except for the overall IPV during pregnancy) and results were described qualitatively. Secondly, the analysis of risk factors for IPV involved tabulating and describing odds ratios or risk ratios with associated 95% confidence intervals and *p*-values. Meta-analysis of risk factors was not possible because the majority of the studies did not report sufficient data for meta-analysis to be performed.

## Results

### Description of studies: design, setting and population

A total of 131 abstracts were identified (see Appendix 1). After screening the abstracts 95 were excluded for not primarily focusing on Africa; research not original and absence of either risk factors or prevalence. A further screening of the remaining 36 papers resulted in further exclusion of another 17 papers because the estimates were not focusing on IPV during pregnancy. Nineteen papers were finally reviewed (see Table 1). Sixteen out of 19 studies employed interviewer administered questionnaires; two used a self administered questionnaire whilst in one study it was not clear how the instrument was administered. Seventeen studies were cross sectional and two used a cohort design. Seventeen were conducted in urban areas while two studies included recruitment from rural areas. Seventeen studies were conducted in a hospital/clinic setting with the majority of women visiting during the antenatal period (14 studies), two studies were conducted in the labour wards, two at the women's own homes and two among women attending postnatal care clinics (some studies recruited from more than one settings).

**Table 1:** Studies reviewed, variables and measurements.

Variables	Ameh et al 2009 [34]	Hoque et al 2009 [35]	Ezechi et al 2009 [36]	Ezechi et al 2004 [37]	Ameh & Abdul 2004 [38]	Chandisarewa et al 2007 [39]	Dunkle et al 2004 [40]	Dunkle et al 2004 [41]	Karamagi et al 2006 [42]	Ntaganira et al 2009 [43]	Ntaganira et al 2008 [44]	Gyuse and Ushie 2009 [45]	Olabuji et al 2009 [46]	Mbokota and Moodley 2003 [47]	Kaye et al 2006 [48]	Fawole et al 2008 [49]	Kaye et al 2002 [50]	Umeora et al 2008 [51]	Efetie and Salami 2007 [52]
Country	Nigeria	South Africa	Nigeria	Nigeria	Nigeria	Zimbabwe	South Africa	South Africa	Uganda	Rwanda	Rwanda	Nigeria	Nigeria	South Africa	Uganda	Nigeria	Uganda	Nigeria	Nigeria
Design	CS	CS	CS	CS	CS	CS	CS	CS	CS	CS	CS	CS	CS	CH	CH	CS	CS	CS	CS
Sample	310	340	652	418	178	221	1366	1395	457	387	600	340	502	570	612	534	379	500	334
Response rate	-	94%	95.5%	80%	65.9%	-	93.1%	95.1%	96%	-	100%	-	-	94.3%	88%	-	-	-	83.5%
Target ppln described?	Y	Y	Y	Y	Y	Y	Y	Y	Y	Y	Y	Y	Y	Y	Y	Y	Y	Y	Y
Sampling	NR	R	NR	-	NR	-	R	R	R	R	NR	NR	-	NR	NR	R	N/R	R	R
Tools used	own	own	WHO	own	own	own	WHO	WHO	own	own	CTS	AAS	WHO	own	AAS	own	AAS	own	own
Case defined	Y	N	Y	Y	N	Y	Y	Y	Y	Y	Y	Y	Y	Y	Y	Y	Y	Y	Y
CI/std errors	N	Y	Y	N	N	N	Y	Y	Y	Y	Y	N	Y	N	Y	Y	N	Y	N
Interview type	intvr	Intvr	self	self	intvr	intvr	intvr	intvr	intvr	intvr	intvr	-	intvr	intvr	intvr	intvr	intvr	intvr	intvr
adjustment	N	N	Y	N	N	N	Y	Y	Y	Y	Y	N	Y	N	Y	Y	N	Y	N
Assessed HIV?	N	Y	Y	N	N	Y	Y	Y	Y	Y	Y	N	Y	N	Y	N	N	N	N
¶ **Overall IPV past 12 months (before pregnancy)** *	-	-	(17% before HIV test)	(39.1%)*	-	-	-	30.1%	-	-	-	-	(43.4%)*	-	-	(14.2%)*	-	-	-
sexual	-	-	-	-	-	-	-	9.7%	65%	-	-	-	-	-	-	-	-	-	-
physical	-	-	-	-	-	-	-	25.5%	14%	-	35.1%	-	-	-	-	-	40.7%	-	-
emotional	-	-	-	-	-	-	-	51%	-	51.9%	-	-	-	-	-	-	-	-	-
¶ **Overall IPV during pregnancy**	28.4%	31%	48.6%	28.7%	28%	8%	-	-	-	-	-	11.6%	28.3%	35%	27.7%	2.3%	57.1%	13.6%	-
sexual	12.9%	15%	-	-	6.1%	-	-	-	-	-	-	-	-	19%	2.7%	-	-	26.5%	-
physical	-	36%	-	-	22.5%	-	-	-	-	-	-	-	-	40%	27.8%	-	-		-
emotional	-	49%	-	-	-	-	-	-	-	-	-	-	-	41%	24.8%	-	-	-	-

**Key**: R = Random; NR = Non Random; Y = Yes, N = No; WHO World Health Organization; CTS Conflict Tactics Scale; AAS = Abuse Assessment Screen; CS = Cross Section; CH = Cohort, intvr = Interviewer administered

*before pregnancy;

¶ **includes all types of violence (physical, emotional, sexual).**

### Quality of studies and risk of bias

Table 2 shows the quality score ranking of studies. The majority (13 or 68%) of studies scored at least five out of the possible eight points whilst three (15.7%) studies scored less than half the possible scores and four (21%) scored half. Two quality measurements that had the least scores (scored less than half) were use of adequate sampling methods and use of validated instruments. The sample sizes in the studies reviewed ranged from 178 to 1395 participants and seventeen out of 19 studies interviewed between 178 and 612 participants. The total number of participants in this review was 8729. [**NB**: Two papers [40], [41] reported from one data set and only the larger sample size was included here]. Eleven out of 19 studies (58%) reported a response rate of at least 80% (eight studies did not report response rates).

**Table 2:** Items used to measure Quality of studies.

Quality item	No. of studies (N = 19)	Percentage (100%)
Use of adequate sampling methods	8	42%
Specification of the target population	19	100
Adequate sample size (≥300)	17	89.4%
Adequate response rate (≥80%)	10	53%
Used known validated and tested tools	8	42%
Reporting confidence intervals or standard errors	11	58%
Adjusting for confounding variables in analysis	10	53%
Attempt to reduce bias	19	100%

Forty-two percent of the studies employed some form of random or systematic sampling whilst the rest employed non-random sampling methods. Most (58%) studies used “own” questionnaires whilst 42% employed commonly used and validated instruments such as the AAS (three studies), WHO questionnaire (four studies) and CTS2 (one study). Fourteen studies reported confidence intervals or standard errors in their analysis of data whilst five presented frequencies only. Ten studies adjusted for different known confounders in their data analysis.

### Prevalence of Intimate Partner Violence in the past 12 months

Four studies reported an overall prevalence of IPV before pregnancy or in the last 12 months. The lowest prevalence reported in these studies was 14.2% whilst the highest prevalence was 43.4%. Prevalence of physical violence in the past 12 months was reported in four studies and ranged from 14% to 41%. See Table 1.

### Prevalence of Intimate Partner Violence during pregnancy

**Figure 1:**
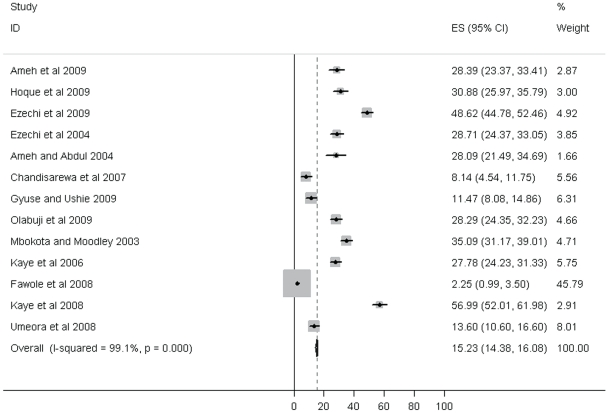
Forest Plot for Overall IPV prevalence.

The overall IPV prevalence during pregnancy was reported in 13 studies (see Table 1). The prevalence ranged from 2.3% to 57.1%. Meta-analysis yielded an overall prevalence of 15.23% (95% CI: 14.38 to 16.08%). See Figure 1 for Forest Plot of Overall IPV Prevalence. There was high heterogeneity between studies (I-squared = 99.1%; *p*-value<0.001). Most (9) of the studies reported prevalences between 27.7% and 51.1% whilst seven reported prevalences between 27.7% and 35%. Sexual violence in the six studies in which this data was clearly presented had a prevalence range of 2.7%–26.5%. Physical violence was reported in four studies and ranged from 22.5% to 40%. Emotional violence was recorded in three studies (24.8%; 41% and 49%).

### Risk Factors for Intimate Partner Violence

#### Low level of education

Only three studies reported strong positive associations between a woman's low level of education and experiencing IPV, that is, Fawole et al [49] (OR 12.54), Hoque et al [35] (OR 7.59) and Umeora et al [51] (*p* = 0.001, OR not stated) whilst in six studies the relationship did not reach statistical significance (*p* = 0.31 in Ezechi et al [36], [37]; *p* = 0.145 in Kaye et al [50]; *p* = 0.05 in Efetie and Salami [52]; *p* value was not stated in Olagbuji et al [46] and Ntaganira et al [44]).

#### Low socio-economic status

In Hoque et al's [35] study it was noted that being unemployed was a risk factor for experiencing abuse (OR 3.57; 95% CI 1.83–6.98) and so was belonging to a low socioeconomic class in studies conducted by Ezechi et al [36] (*p* = 0.000) and Umeora et al [51] (*p* = 0.0037) and having less household decision-making power (*p* = 0.009) in Kaye et al [48], [50]. There was no difference in the experience of abuse between women who were unemployed and those who were employed in either skilled or informal sector (p = 0.701) in Kaye et al [50].

#### Young age

Five studies reported on the relationship between age and experiences of abuse among pregnant women with three studies reporting significant associations [44], [49], [50] and two reporting no associations (*p* = 0.45 in Ezechi et al [37], and *p* = 0.11 in Ezechi et al [36]. Younger age such as being an adolescent compared to non-adolescent (over 20 years) were found to be associated with abuse (*p* = 000) in Kaye et al [50] and Fawole et al [49] while in Ntaganira et al [44] being a young adult (26–34 years) was associated with experiencing abuse compared to those aged between 18 and 25 years (OR 1.35).

#### HIV Diagnosis

Of the 19 studies reviewed 10 (52.6%) collected data on HIV prevalence or knowledge of serostatus among pregnant women. Table 3 shows the relationship between HIV and IPV. After adjustment for known confounders, five studies retained a positive association between HIV and IPV during pregnancy. These studies showed that being diagnosed for HIV or testing HIV positive increases pregnant women's chances of being abused by a partner. The increase in likelihood of a HIV infection ranged from a minimum OR of 1.48 to a maximum OR of 3.1. Three studies did not find a significant association and two did not test/report findings on relationship between IPV and HIV.

**Table 3:** Relationship between HIV and IPV during pregnancy.

Author	Variable related to IPV	Measurement	HIV status check
Dunkle et al (2004) [40]	HIV positivity	*p* = 0.002; OR 1.48 95% CI 1.15–1.89	Determine Rapid and Capillus tests
Ezechi et al (2009)	HIV negativity of spouse (study was done among HIV+ women)*	*p* = 0.001; OR 3.1 95% CI 2.4–5.3	Laboratory HIV test for women and women's report for spouses' status
Hoque et al (2003)	Knowing own HIV status	*p* = 0.000 OR 2.93 95% CI 1.79–4.81	Self reported
Olagbuji et al (2010)	HIV positivity	*p* = 0.02, OR 2.81 95% CI, 1.2–6.5	Self reported
Ntaganira et al (2008)	HIV positivity	*p*<0.001;OR 2.38 95% CI 1.59–3.57	ANC clinic records
Ntaganira et al (2009)	HIV positivity	*p* value not stated (non-significant) OR 1.06 95% CI 0.66, 1.73	ANC records
Kaye et al (2006)	HIV positivity	*p*-value not stated (non significant)	Not reported
Karamagi et al (2006)	HIV test last pregnancy	*p*-value not stated (non-significant) OR 1.8, 95% CI 0.6–5.3	Self reported
	HIV talk with husband	*p* value not stated (non-significant) OR 1.6, 95% CI 1.0–2.6	Self reported

*Comparison group was non-abused women; *p* = *p* value; OR = Odds Ratio; CI = Confidence Interval.

#### Sexual Risk factors

In multiple variable logistic regression models, sexual risk factors positively associated with experiencing IPV include transactional sex and having more than 5 lifetime sexual partners (OR 1.69; 95% CI 1.21–2.37) [40]; having a partner with multiple sexual partners (OR 1.53; 95% CI 1.15–2.20 and OR 3.2; 95% CI 2.0–5.0) in Ntaganira et al's [44] and Karamagi et al's [42] studies respectively and having sex with another man whilst in marriage (OR 2.8; 95% CI 1.0–7.7) in Karamagi et al's study [42]. However, condom use by a partner was not significantly (OR 1.2, 95% CI 0.7–2.3) associated with IPV [42] (data not shown).

#### History of violence

There is strong evidence from seven studies that a history of abuse (defined as experiencing abuse before the age of 15, abuse in the past 12 months and abuse in lifetime) is significantly associated with IPV in pregnancy or just before pregnancy as shown on Table 4. Of these studies, only three showed observed statistical differences between history of violence and current violence during pregnancy (*p*≤0.023) but did not show risk or odds ratios.

**Table 4:** Relationship between history of violence and IPV during pregnancy.

Author	Variable related to IPV during pregnancy	Measurement
Dunkle et al (2004) [40]	Child sexual abuse	RR 2.43; 95%CI 1.93–3.06
	Forced first sexual intercourse	RR 2.64; 95%CI 2.07–3.38
Kaye et al (2002)	Witnessing abuse in childhood	*p* = 0.000
	Physical abuse in childhood	*p* = 0.023
Ntaganira et al (2008)	Abuse in childhood	OR 2.69; 95%CI 1.69–4.29
Ntaganira et al (2009)	Any form of violence	*p* = 0.0001
Olagbuji et al (2010)	IPV 12 months before pregnancy	*p*<0.0001 OR 274.34; 95% CI 66.4–1133.8
Karamagi et al (2006)	Sexual violence	OR 3.7; 95% CI 2.1–6.6
Ezechi et al (2009)	Abuse before HIV test	*p* = 0.003

*p* = *p* value; CI = Confidence Interval; OR = Odds Ratio RR = Risk Ratio.

#### Alcohol use

Five studies examined the relationship between alcohol use and IPV and all of them found that alcohol use by a woman and/or partner whether heavily or occasionally is significantly associated with pregnancy-related abuse. See Table 5.

**Table 5:** Relationship between alcohol use and IPV during pregnancy.

Author	Variable related to IPV	Measurement
Dunkle et al 2004 [40]	Woman's alcohol/drug problem	*p* = 0.0002 OR 4.59; 95%CI 2.54–8.30
Olagbuji et al 2010	Woman regularly takes alcohol	*p*<0.0001 OR 11.60; 95% CI 3.8–35.1
Ntaganira et al 2008	Partner heavily drinks alcohol	*p* = 0.0001 OR 3.37; 95% CI 2.05–5.54
	Partner occasionally drinks alcohol	OR 4.10 95% CI 2.48–6.77
Ntaganira et al 2009	Partner occasionally drinks alcohol	OR 2.52 95% CI 1.35–4.71
	Partner heavily drinks alcohol	OR 3.85; 95% CI 1.81–8.21
Fawole et al 2008*	Partner drinks alcohol	*p*<0.001; OR 2.89; 95% CI 1.51–5.53

*p* = *p* value; OR = Odds Ratio; CI = confidence Interval;

*alcohol abuse was related to IPV 12 months before pregnancy.

## Discussion

The review found a wide range in the overall prevalence of IPV during pregnancy ranging from as low as 2% to as high as 57%. This wide range is somewhat similar to what was reported in Gazmararian et al's review [2] (0.9–20%) and Taillieu and Brownridge [3] review (0.9–30%). Similarly, the WHO [28] Multicountry study that collected data from 10 countries reported IPV prevalence during at least one pregnancy ranging from 1% to 28%. The disparities in our review can be explained in two ways. Firstly this could be attributed to methodological differences across studies. The lower prevalence in some studies is very likely due to methodological limitations. For instance Fawole et al's study [49] which reported the lowest rate (2.3%) excluded women who if included, could have contributed to a higher and more accurate prevalence. The authors mentioned that, “Women who expressed fear that granting the interviews may result in further violence were excluded from the interviews” [49] Although the number of women excluded for this reason was not mentioned it clearly shows that the excluded women resulted in underreporting and lower estimates. In addition, the study used own tool with few semi-structured questions. The author's non-reporting of response rate was another limitation of the paper. It was this outlier during meta-analysis that contributed the most weighting (45%) (Figure 1) leading to higher heterogeneity. Other studies which reported lower prevalence (8.3%, 11.6%), used own tools or AAS in the case of Chandisarewa et al [39] and Gyuse and Ushie [45] respectively or tools with few items measuring violence (13.6%) in the case of Umeora et al [51]. Taillieu and Brownridge [3] also concluded that methodological issues influenced disclosure. Such under-reporting rather than over-reporting has been identified in violence against women studies in general [53].

Secondly, despite the methodological limitations in a few studies, the great disparities could be showing real differences in levels of occurrences of violent acts in African regions and cultural groups. The fact that most of the studies (9 out of 13) show prevalences above 27% means that the prevalence of violence during pregnancy is very high in Africa. This is similar to trends of violence among women in the general population in Africa [5]. Such high prevalences could be a result of gender inequalities organized mostly around patriarchal lines in Africa [54]. However, qualitative studies are needed to explore such dynamics and disparities in prevalence figures in general and among pregnant women. Another possible explanation for the higher levels could be related to greater reporting of violence due to increased use of tested instruments. This was a recommendation from Gazmararian et al [2] that the use of validated instruments could result in more disclosure of violence.

Since most of the studies on violence against women are cross sectional in design, there is a dearth of literature on violence trends before pregnancy, during pregnancy trimesters and after pregnancy. There is some evidence in this review that violence decreases during pregnancy by at least 10%. Only four studies measured prevalence of violence both before and during pregnancy. Three of these studies show that prevalence of violence during pregnancy was lower than violence in the past 12 months or before pregnancy. Olagbuji et al [46] reported 43.4% and 28.3% before and during pregnancy respectively, whilst Fawole et al [49] reported 14.2% and 2.3% before and during pregnancy respectively and Ezechi et al [37] reported 39.1% and 28.7% before and during pregnancy respectively (Table 1). The same trend has been observed in other parts of the world [10], [55]. This possibly shows the protective effect of pregnancy against IPV and requires further exploration to understand the socio-cultural factors that influence the decrease of abuse during pregnancy.

The absence of data on the association between HIV testing and abuse during pregnancy meant that conclusions could not be drawn. Only one study [36] demonstrated that; before testing for HIV the prevalence of IPV was 17% and after testing for HIV and disclosing their status 62.7% reported being abused by their partners. Chandisarewa et al [39] showed that 8% were abused after testing for HIV but did not give a baseline figure to show the proportion of pregnant women who were abused before HIV test. A larger cohort study will be needed to observe trends in IPV before and after HIV testing in a pregnant population to understand the effect of disclosure of HIV status on IPV. Such research is crucial for the development of health services intervention such as screening for IPV during HIV testing during pregnancy and providing support to pregnant women.

This review has shown that HIV diagnosis and seropositivity are positively associated with experiencing IPV during pregnancy. This was found in five studies and reflects what has been reported in the general population as well [20], [25]. Evidence of the interconnections between HIV and IPV has been demonstrated by the IMAGE study [56] and Stepping Stones study [57] in South Africa where interventions in gender and IPV training reduced HIV sexual risk factors. This association with HIV status could be related to the increase in HIV screening which is almost becoming universal among pregnant women through the provider initiated HIV testing in most countries. All countries in which the studies in this review were conducted are in the Sub-Saharan region which records the highest prevalence of HIV among women of childbearing age in the world [58]. We need to understand how HIV status operates in a culture where female subordination is the norm and how together with other factors it increases pregnant women's risk for violence.

It is clear from the study that abuse of alcohol or drugs by partner (or self) is a risk factor for being abused by a partner. Results in this review are consistent with results across the world [3] in that alcohol and drug abuse are significantly associated with partner violence. The higher odds ratios obtained in the studies reviewed on the relationship between alcohol or drug use and IPV could have been influenced by how the instruments were used to measure alcohol use. For example, in a study by Olagbuji et al [46] which reported the highest odds ratios (OR 11.60; 95% CI 3.8–35.1) the question on alcohol abuse was too general; researchers asked if the respondent had taken “1 or more alcoholic drinks per month in the last 3 months” and this was coded regular alcohol use if a respondent answered affirmatively. This overestimated the strength of the relationship with partner violence. Whilst Ntaganira et al [43], [44] asked if a respondent's partner used alcohol sometimes, frequently/always or never, Dunkle et al [40] asked if a respondent ever had a fight, accident, injury, casual sex, or got arrested after drinking to assess problem drinking. Whilst all the other studies assessed either frequency or effects of alcohol intake, Fawole et al [49] only assessed whether partner or respondent took alcohol or not. This raises issues of measurement bias since alcohol intake was not clearly defined; respondents taking one drink were similarly considered with those who drank to intoxication and therefore possibly exaggerating the magnitude of association with IPV. There is need to use validated measures of alcohol abuse to avoid overestimating the strength of the relationship.

The review showed a strong relationship between a history of violence and current violence in pregnancy although the range and types of violence varied including child abuse and previous year experience of violence among pregnant women. Reviews elsewhere demonstrated that adult women (though not pregnant) with a history of childhood sexual abuse show stronger evidence of revictimisation than non-abused women [16], [17], [18]. One explanation put forward is that when women are abused in childhood they learn that subordination to males and experiencing violence are part of being a woman. They become vulnerable and therefore depend on men [17]. This may hold true in the context of IPV during pregnancy when women are less able to economically protect themselves. Being younger and having low socio-economic status compared to their partners may also contribute to them being abused by their partners who are older and have economic power and security. Since low socio-economic status is linked with being abused, it would therefore imply that raising women's income levels through access to and control of economic and financial resources could significantly lower their chances of being abused. In the IMAGE study in South Africa women who were economically empowered through credit extension and managing loans reported reduced risk of IPV [56].

### Strengths and weaknesses

Most of the studies scored above average on the study quality score. The quality of the studies was increased by the fact that most controlled for confounding variables in the multivariate logistic analysis. However, sample sizes in the studies were generally low and the use of standardized and validated instruments was low. The review did not look at clinical outcomes of abused women during pregnancy. Such an analysis of clinical outcomes could help to further influence policies on screening and other interventions at the health system level. An analysis of some questions of violence in studies which used own tools shows some resemblance of the McFarlane et al's [31] Abuse Assessment Screen (AAS) which over the years has influenced clinical assessments and research in gynecological settings despite its limitations such as its short length, combined items for measuring physical and sexual violence, non-availability of any measure of emotional violence and its use of words such as “abuse” in asking violence questions instead of behavioral acts such as used in the WHO [28] questionnaire and the Conflict Tactics Scale 2 (CTS2) [30]. The comparison between the AAS and the CTS2 has been done elsewhere [59] and results show less reliability in the AAS.

### Conclusion

This review contributes knowledge of prevalence of and risk factors for IPV during pregnancy in Africa and shows clear evidence that the prevalence of IPV is very high in pregnant women on the continent. The major risk factors for IPV are alcohol and drug use, sexual risk taking, HIV infection and a history of violence and points to the need for interventions with pregnant women as part of antenatal care. Such screening and programs should address both prevention of IPV and HIV since it essentially deals with similar women empowerment issues.

## Supporting Information

Appendix S1 **Flow Diagram**
(DOCX)
