# Supplementary material for: A Systematic Review of African Studies on Intimate Partner Violence against Pregnant Women: Prevalence and Risk Factors
Source: PLoS One. 2011 Mar 8;6(3):e17591. doi: 10.1371/journal.pone.0017591 (PMC3050907; doi:10.1371/journal.pone.0017591)
Supplement: Appendix S1 — Flow Diagram (DOCX) [file pone.0017591.s001.docx]

**Appendix 1: Flow Diagram**

Records screened
(n = 131)

Records after duplicates removed
(n = 131)

## Identification

Records identified through database searching (n = 103)

Additional records identified through other sources (n =28)

Full-text articles excluded- no estimates (n =17)

Records excluded (qualitative, research not original, not primarily focussing on Africa/pregnancy
(n = 95)

## Screening

## Eligibility

## Included

Studies included in quantitative synthesis (meta-analysis) (n = 19)

Full-text articles assessed for eligibility (n = 36)
